# Effects of Arctigenin in Proliferation, Migration, and Invasion of Nasopharyngeal Carcinoma 5-8F Cells

**DOI:** 10.2174/1871520623666230228155129

**Published:** 2023-06-01

**Authors:** Dongdong Huang, Rui Lu, Mingjing Cai, Jie Meng, Shuangba He, Qingxiang Zhang, Wei Meng

**Affiliations:** 1 Department of Otorhinolaryngology-Head and Neck Surgery, Nanjing Tongren Hospital Affiliated to Medical College of Southeast University, Nanjing, Jiangsu Province, 210000, China;; 2 Ophthalmology Department, Affiliated Eye Hospital of Nanjing Medical University, Nanjing, Jiangsu Province, 210000, China

**Keywords:** Arctigenin, nasopharyngeal carcinoma, migration, invasion, proliferation, western blotting

## Abstract

**Background:**

Nasopharyngeal carcinoma (NPC) is a malignant tumor of the nasopharynx.

**Objective:**

Here, we aimed to understand better the molecular basis for arctigenin (ARG)’s ability to promote NPC 5-8F cell invasion.

**Methods:**

We tested the effects of several doses of ARG on 5-8F cells that had been cultured *in vitro*. We estimated the metabolic activity of cells by The MTT (3-(4,5-dimethylthiazol-2-yl)-2,5-diphenyltetrazolium bromide) tetrazolium assay. We examined the influence on cell invasion, and migration using Transwell Evaluation. Real-time polymerase chain reaction analysis was used to determine the relative amounts of epidermal growth factor receptor (EGFR), Janus kinase 2 (JAK2), and transcriptional activator 3 (STAT 3) mRNA expression. Using western blotting, we looked at the level of phosphorylation of specific proteins like EGFR, phosphorylated EGFR, JAK2, and STAT 3.

**Results:**

Our findings revealed that ARG inhibited NPC 5-8F cell development in a dose-and time-dependent manner. The invasiveness and mobility of 5-8F cells were significantly suppressed when ARG was overexpressed in a tumor development model. Expression levels of EGFR, JAK2, and STAT 3 mRNA were considerably low in the experimental group. As a consequence of being treated with ARG, lower levels of EGFR, p-EGFR, p-JAK2, and p-STAT3 expression were observed.

**Conclusion:**

These results suggest that ARG may prevent NPC 5-8F cells from proliferating, migrating, and invading other tissues. There are a few potential molecular pathways, two of which are the inhibition of EGFR phosphorylation and the reduction of levels of phospho-JAK2 and phospho-STAT3.

## 1 INTRODUCTION

Malignant tumors of the nasopharynx are known as nasopharyngeal carcinoma (NPC), and their development is strongly linked to the Epstein-Barr virus. Although the global incidence rate is low, high-incidence regions are primarily found in Southeast Asia and southern China, with rates as high as 15–50/100,000 in some areas of south China [1]. Since the nasopharynx is usually out of sight, most people with NPC are diagnosed at a late stage (stages III or IV) when they first show symptoms. The squamous cell carcinoma subtype to which NPCs most often belong is particularly aggressive and prone to metastasize to other parts of the body. Surgical resection is challenging for NPCs because the origin site is deep and in close proximity to many vital organs. Despite their prevalence, radiotherapy and chemotherapy continue to be the primary treatments for NPCs, despite their relatively severe side effects and limited clinical therapeutic effects [2, 3]. The leading causes of death in NPCs are local recurrence and distant metastasis due to radiotherapy resistace. The primary difficulty in enhancing NPC's therapeutic efficacy and prognosis is the effective inhibition of recurrence and metastasis. Chinese medicine's low rate of adverse reactions and significant efficacy in the prevention and treatment of tumors have attracted scholarly attention in China and around the world in recent years.

*Fructus arctii*, the dried and mature fruit of the plant species *Arctium lappa* L. from the family Compositae, has been used for centuries to treat various ailments, including wind-heat, phlegm in the lungs, sore throat, rash, detoxification, and detumescence. Its bitter flavor and chilliness cause it to travel through the lungs and stomach meridian channels. *Fructus arctii* contains Arctiin and arctigenin (ARG), the latter of which is the decomposition product of Arctiin [4]. Several studies have shown that arctigenin has significant pharmacological effects. The body's immune response can be controlled by arctigenin's ability to modulate the response mechanism of immune factors, and arctigenin can also inhibit the inflammatory response by controlling the response mechanism of inflammatory factors. Treatment with arctigenin can fortify the immune system to better withstand the virus. As previously mentioned, arctigenin is useful in treating kidney disease and tumors [5-8]. Additionally, Arctii root glucoside (ARG) has been shown to improve chemotherapeutic response rates [9]. Several cancers, such as those of the gallbladder, stomach, lungs, and prostate, are treated with ARG [10-13].

To kill cancer cells, Gu *et al*. (2012). discovered that ARG inhibits mitochondrial respiration when glucose is scarce, decreasing intracellular ATP and increasing reactive oxygen species [14]. *In vitro* studies, Susanti *et al*. (2013) discovered that ARG treatment of lung adenocarcinoma cells significantly stalled their growth cycle, keeping them predominantly in the GO/G1 phase of the cell cycle and reducing their expression of the NPAT protein. Lung adenocarcinoma cell growth may be suppressed by arctigenin because of the compound's potential to regulate NPAT protein expression, thereby reducing cellular proliferation-inhibiting -dependent kinase 2 or 7 [15]. Yang *et al*. (2012) studied bladder cancer T24 cells and showed that ARG could selectively alter the phosphorylation level of members of the mitogen-activated protein kinase (MAPK) protein family by regulating ERK1/2 phosphorylation and up-regulating P38 phosphorylation level, thereby inducing tumor cell apoptosis [16]. Researchers in this study used the NPC 5-8F cell line as a model to learn more about the molecular mechanism by which ARG affects the proliferation, migration, and invasion of 5-8F cells and to generate novel strategies for the all-encompassing treatment of NPC.

## 2 MATERIALS AND METHODS

### 2.1 Experimental Cells and Reagents

Human cell line 5-8F NPC (Shanghai Cell Bank, Chinese Academy of Sciences), ARG (Sigma, China), Roswell Park Memorial Institute (RPMI) 1640 medium, PBS buffer, bovine serum, fetal bovine serum (FBS), trypsin- EDTA solution (Gibco), Transwell cells (Corning), RNA extraction reagent (Thermo Fisher), first strand cDNA synthesis kit, Real Mastermix (SYPR Green Co., Ltd.), real-time PCR kit (Diangan Co., Ltd.)., reverse transcription-polymerase chain reaction (RT-PCR) primers (Shanghai Shengkong), rabbit monoclonal antibodies based on glyceraldehyde-3-phosphate dehydrogenase (GAPDH), EGFR, p-EGFR, p-JAK2 and p-STAT3 (Cell Marker), ECL electrolyte (Millipore), MTT reagent, dimethyl sulfoxide (DMSO), and goat anti-rabbit IgG-HRP (Solubisoft, Beijing).

### 2.2 Cell Treatment and Transfection

NPC 5-8F cell cultures were prepared in the laboratory. They were cultured and incubated after being seeded in RMPI-1640 media with 10% FBS. The incubator was set at the optimal conditions of 37°C temperature, 5% carbon dioxide, and 95% humidity. 5-8F cells develop in an adherent manner, which can be seen by researchers during the culture phase. Every two days, on average, the medium in the culture was changed. The cells were subcultured at a 1:3 ratio, and those with logarithmic growth rates were harvested for analysis.

### 2.3 Experimental Groups and Drug Configurations

The ARG was dissolved in DMSO solution to make a mother liquor at a 100 × 10^3^ mol/L concentration. A dark refrigerator set to -20°C was used to keep the mother liquor from spoiling. To find out how much drug would be needed for the experiment, the mother liquor was diluted with RPMI1640 culture solution from a concentration of 0 mol/L to a final concentration of 80 mol/L. No concentration of the experimental medicine included more than 0.1% DMSO.

### 2.4 Cell Proliferation Assay

When the 5-8F cells reached the logarithmic phase, they were collected. Adjusting the cell density to 4 × 10^5^ cells/mL required adding trypsin with 0.25% EDTA for digestion and a sufficient volume of PBS for dilution. 100 µL of the cell suspension was plated into each well of a 96-well plate. After some time in a CO_2_ incubator, the cells attached to the surfaces. To compare the effects of various ARG concentrations (0, 20, 40, and 80 mol/L), the medium containing these concentrations was swapped out for a 0 mol/L control group, and an additional three wells were added to each group. After a predetermined incubation duration of 24 h, 20 µL of MTT solution (5 mg/mL with PBS) was added to each well, and the plates were incubated for another 4 hours to meet the experimental conditions. Once the supernatant was drained off, the culture was thrown out. Each well was treated with (DMSO). After vigorous shaking, the crystals were entirely disintegrated. The percentage of cell growth inhibition was determined by measuring each well's optical density using a microplate reader at 570 nm.

### 2.5 Transwell Migration Assay

5-8F cells were exposed to several doses of ARG (0, 20, 40, and 80 mol/L), with a concentration of 0 mol/L as control. The 5-8F test cells were grown to the logarithmic phase, digested, washed in PBS and serum-free media, counted, suspended in serum-free medium, and diluted to 2 × 10^5^/mL. The three wells in the top chamber of the Transwell were punctured, and 200 µL cell suspensions were injected from each group. Next, 500 µL of fetal bovine serum–supplemented Dulbecco’s Modified Eagle Medium (DMEM) was poured into the Transwell chamber's bottom layer. Cells were cultured in a cell incubator at 37°C for 24 hours before being withdrawn from the chamber, having their medium sucked out of the top chamber, and being collected with a cotton swab from the membrane filter, where they had not gone through. The microscopical inspection was used to determine the total number of cells on the underside of the PET film after they were fixed in a 70% methanol solution for 15 minutes and then stained with crystal violet or trypan blue for 20 minutes.

### 2.6 Transwell Invasion Assay

A layer of Matrigel gel was placed in the top chamber, and cells from each treatment group were inserted into the gel. The other treatments were just like the ones used in the Transwell migration study. Microscopical analysis was used to determine the total number of invading cells.

### 2.7 Real-time PCR Assay

For 24 hours after treatment, 5-8F cells were seeded at a density of 4, 105 cells per well in a 6-well plate. To get to the total RNA in the cells, the TRIzol method was utilized. The spectrophotometer was calibrated using an RNA solution diluted with TRIS-EDTA buffer, and the solution's concentration and purity were read off from the absorbance values at 260 nm and 280 nm. After RNA was converted to cDNA, PCR was carried out using the instructions provided with the SYBR Green fluorescent dye kit. PCR products were electrophoretically separated from the DNA ladder on an agarose gel containing 2% of the reaction mixture. To see whether the PCR products separated into distinct amplification bands, the gel was stained with gold view. At the time the PCR amplification reaction was beginning, the prepared PCR reaction solution was added to the real-time PCR machine. Pre-denaturation was finished after 2 minutes at 93°C. The response time was as follows: 1 minute at 55°C, 1 minute at 72°C, and 7 minutes at 72°C (for a total of 40 cycles). Determined primer sequences are shown in Table **1**. Altogether, there might be three compound holes in each cluster. Each well's CT value or cycle threshold was documented. Gene expression levels were quantified using the 2-Ct method.

### 2.8 Western Blotting

5-8F cells were lysed for each treatment group to collect proteins. The Bradford assay was used to determine protein content. After the proteins were separated using SDS-PAGE, they were transferred to a PVDF membrane using the same amount of protein. The membrane was then blocked in 5% non-fat dried milk for an hour and incubated at 4°C for the night. After incubating the primary antibody for 4 hours at a temperature of 4°C, the secondary antibody was incubated for 1 hour at room temperature. The sample was then subjected to enhanced chemiluminescence (ECL) liquid. The primary antibody dilution ratios were Goat anti-rabbit (GAPDH), diluted 1:1000; epidermal growth factor receptor (EGFR), diluted 1:1000; phospho-EGFR, diluted 1:1000; phospho-JAK2, diluted 1:1000; and phospho-STAT 3, diluted 1:1000. A concentration of 1:10000 was used for the dilution of the secondary antibodies. The percentage of deviation from the GAPDH internal standard was determined by comparing the outcomes of three separate tests carried out on each sample. These tests were carried out independently.

### 2.9 Statistical Analysis

The statistical analysis of the data was performed with the help of the SPSS program (version 19.0). All data are reported as the mean data ± standard error of the mean (SEM). The average of the various sources was compared using one-factor analysis of variance. Pairwise comparisons were made using the LSD and SNK tests. T-test was used to evaluate the central tendency of the two samples. Results with a probability of less than 0.05 were deemed significant.

## 3 RESULTS

### 3.1 ARG's Impact on 5-8F Cell Proliferation

**Fig. (1):**
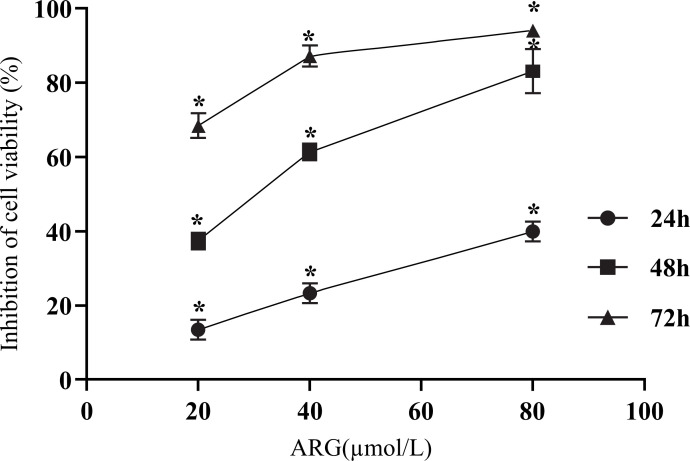
ARG inhibited the proliferation of 5-8F cells. 5-8F cells were treated with various ARG concentrations (0, 20, 40, and 80 mol/L). Control cells were treated with 0 mol/L ARG. Cell viability was assessed by the MTT assay. The data are presented as meanRG. C of six separate experiments. *P < 0.05.

According to the MTT test findings, ARG could reduce the viability of 5-8F cells when they were cultured *in vitro*. When compared to the control group, the effects of ARG on the growth of 5-8F cells were considerably inhibited, and the magnitude of this impact was dose-dependent. Meanwhile, it was demonstrated that the inhibitory impact became noticeably more pronounced as the action period further stretched out. SPSS determined that an IC50 of 38.91 mol/L was reached after being exposed to ARG for 48 hours (*P <* 0.05; Fig. **1**).

### 3.2 The Impact of ARG on 5-8F Cell Migration and Invasion

**Fig. (2):**
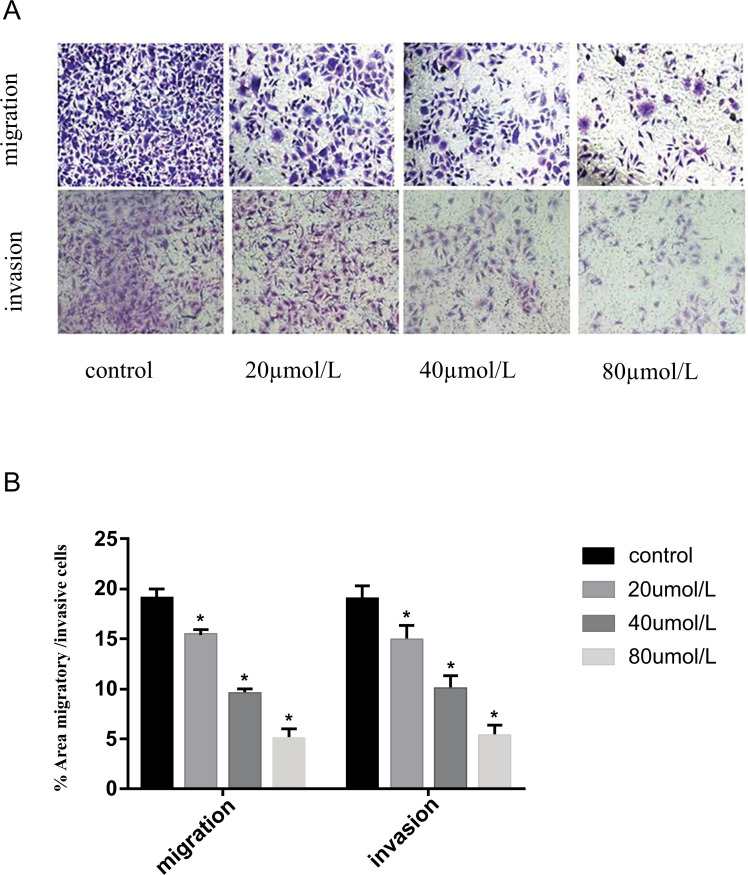
Effect of ARG on the migration and invasion of 5-8F cells. (**A**) 5-8F cells were subjected to transwell assay for migration and invasion assay. (**B**) Data are presented as mean invasion assay. assay. gration and *P* < 0.05.

Results from Transwell invasion and migration experiments showed that ARG treatment at concentrations of 20-40-80 mol/L significantly reduced 5-8F cell migration and invasion compared to untreated controls (0 mol/L ARG). 5-8F cell migration and invasion were inhibited inversely as a function of ARG concentration (*P <* 0.05; Fig. **2**).

### 3.3 The Role of ARG in Regulating Related mRNA Expression in 5-8F Cells

**Fig. (3):**
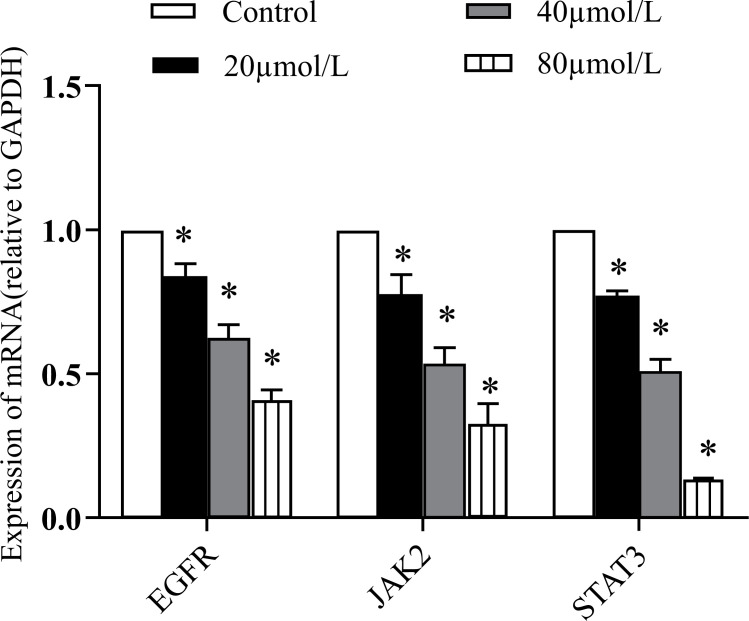
Effects of ARG on mRNA expressions in 5-8F cells. 5-8F cells were treated with 20, 40, and 80 mol/L ARG. Data are presented as mean and 80 mol/L ARG. Data *P* < 0.05.

A series of RT-PCR experiments were performed to quantify mRNA expression. After being exposed to several medication concentrations, EGFR, JAK2, and STAT3 mRNA expressions in 5-8F cells were drastically reduced (*P <* 0.05; Fig. **3**). This was true after exposure to ARG at concentrations of 20, 40, and 80 mol/L.

### 3.4 Effects of Protein Expression on the ARG Signaling Pathway

**Fig. (4):**
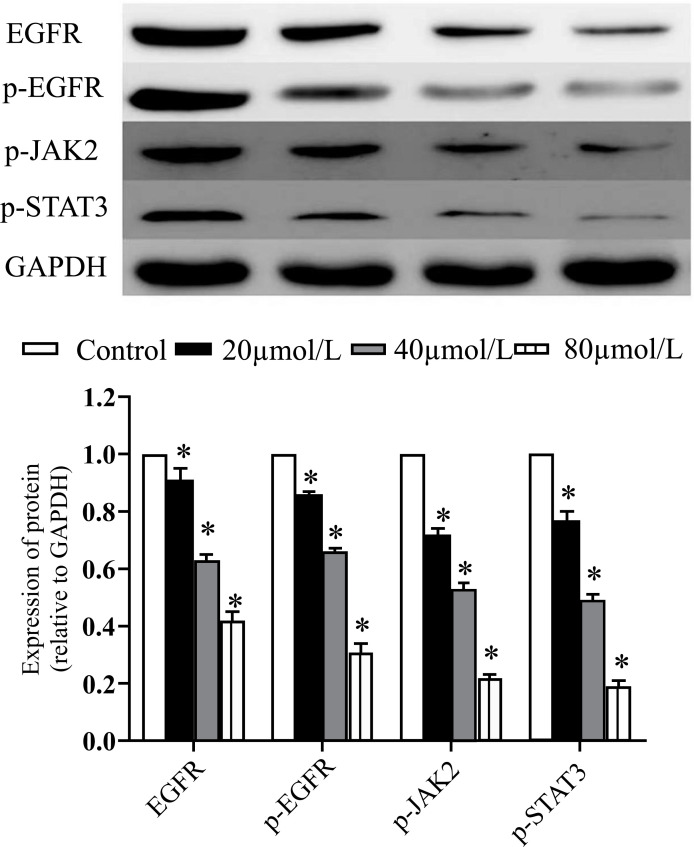
Effects of ARG on protein expressions in 5-8F cells. 5-8F cells were treated with 20, 40, and 80 mol/L ARG. The bands were quantified using Image-Pro Plus software. The results shown are representative of three independent experiments. Results are expressed as the mean pressed *P* < 0.05.

We used western blotting to examine protein expression and discovered that ARG downregulated the activity of EGFR, p-EGFR, p-JAK2, and p-STAT3. Protein expression levels for EGFR, p-EGFR, p-JAK2, and p-STAT3 dropped when 5-8F cells were treated with 20, 40, and 80 mol/L ARG, respectively (*P <* 0.05;Fig. **4**).

## 4 DISCUSSION

Compared to the local head and neck surgery rate, the incidence of NPC is much greater in Southern China. Because of its anatomical position and radiosensitivity, radiotherapy is the primary treatment for NPC. But radiation resistance and its possible adverse effects are significant reasons for treatment failure. Traditional Chinese medicine (TCM) is gaining popularity as a viable option for cancer therapy because of its positive characteristics, including low toxicity, high efficiency, and ability to attack numerous cancer-related pathways simultaneously. Modern studies aim to discover new and more efficient anticancer medicines derived from TCM. It has been shown that TCM may enhance the quality of life, raise immunity, and lengthen the lifespan of patients with cancer undergoing chemotherapy, radiation, or both. Many researchers, both in China and abroad, are looking for effective natural medications or their active components to combat NPC. Homologous NPC with varying degrees of radiation resistance may be differentiated and induced to undergo apoptosis with the use of the curcumin derivative T83. Researchers have discovered that exposure to tetrandrine makes human nasopharyngeal cancer cell lines more susceptible to radiation therapy.

ARG is a crucial lignan extract according to TCM. According to research, tumor development and death may be mediated by ARG [7, 17-21]. ARG does this by interfering with the cell cycle, halting cell proliferation, and preventing tumor angiogenesis. Few studies on how ARG affects NPC cells exist. Based on the results of the MTT assay, we found that ARG dramatically decreased the viability of NPC 5-8F cells. Furthermore, the dosage and time required for ARG to exert its inhibitory impact on cells increased with increasing drug concentration, indicating that ARG was both times- and dose-dependent in its inhibition of 5-8F cells. After 48 hours of treatment with ARG, the IC_50_ was determined to be 38.91 mol/L using SPSS.

Both the survival and expansion of many types of cancer cells depend on the JAK2/STAT3 signaling pathway [22]. Cancer cells are able to evade immune-cell-induced apoptosis because of the activation of downstream proteins, such as those involved in the cell cycle, survival, and anti-apoptotic proteins. When activated, STAT3 promotes tumor development and progression [23]. Extensive studies have shown that STAT3, a protein related to STAT, is commonly activated in cancer. Compared to other STAT family members, STAT3 is highly phosphorylated and may promote tumor development. According to a prominent study team, blocking STAT3 reduces tumor development with little impact on healthy cells. These results support STAT3 as a viable therapeutic target for cancer management. STAT3, formerly considered a transcription factor, has also been shown to have essential cellular processes.

Initiation, development, and spread of tumors are all influenced in various ways by the abovementioned elements. Aberrant STAT3 activity is linked to a wide variety of solid and hematologic malignancies, including colon, lung, and prostate cancers, and is most often caused by disruptions in cytokine signaling [24]. Consistent STAT3 activation in melanoma results from SRC and JAK activation [25]. Melanoma cell growth and development are stimulated by aberrant STAT3 expression. Abnormal STAT3 activation in prostate cancer cells is linked to EGF receptor and JAK kinase-mediated trafficking of IL-6 and IL-11 cytokines [26]. STAT3 is being investigated for its potential involvement in prostate cancer. STAT3 has been shown to increase cell proliferation and cell cycle in prostate cancer [27, 28], hence promoting cell survival. Tumors of the pancreas, lungs, kidneys, esophagus, cervix, colon, and gastrointestinal tract benefit from aberrant STAT3 activity, which also enhances tumor cell survival and adds to the malignant phenotype. It has been shown that active growth factor receptors and non-receptor tyrosine kinases are associated with prolonged STAT3 activation in non-small cell lung cancer [29]. Pancreatic, ovarian, and colon cancers have been associated with VEGF, EGFR, SRC, and JAK overexpression and STAT3 activation. The survival of tumor cells depends on STAT3's ability to activate MCL-1 [30].

STAT3 is a tyrosine kinase that functions within the cell and is activated when it is phosphorylated by JAK2. After being activated, STAT3 forms a homodimer moves into the nucleus and controls gene expression [31]. It helps spread cancer and encourages the development of malignant cells. Using real-time PCR, we discovered that ARG-treated 5-8F cells had increased mRNA expression of the JAK2/STAT3 signaling pathway. The western blotting analysis further showed that ARG therapy led to a reduction in JAK2/STAT3 protein expression. Hepatocellular carcinoma tumor metastasis and epithelial-mesenchymal transition characteristics are both suppressed by ARG since this protein has been found to disrupt the Wnt/-catenin signaling pathway [32]. Also, ARG may suppress glioblastoma development by activating autophagy through the AKT/mTOR pathway [33]. These results suggest that ARG regulates JAK2 and STAT3 expression to suppress tumor cell proliferation, invasion, and metastasis. All of the nasopharyngeal carcinoma cells analyzed here were cultured in a lab. Additional research into the efficacy of ARG and other molecular pathways *in vivo* and *in vitro* is required.

## CONCLUSION

Following *in vitro* cultivation in a variety of experimental circumstances, this research uncovered shared biological features among NPC 5-8F cells. Inhibition of the JAK/STAT signaling pathway was validated, demonstrating that ARG might reduce NPC 5-8F cell proliferation, migration, and invasion to display antitumor effects. ARG's experimental and clinical use for NPC therapy will benefit from a better understanding of these pathways.

## Figures and Tables

**Table 1:** RT-PCR primer sequences.

**Gene**	**Primer (5’-3’)**	**Product Size/bp**
*EGFR*	F: ATGAAAACACCTATGCCTTAGCC	83
	R: CATCCCCTCCCGTTTCTTCTTT	
*JAK2*	F: GGGTGGAGACAACTGTGACG	178
	R: GAAGTGGCGACGCGAACA	
*STAT3*	F: CATCCTGAAGCTGACCCAGG	183
	R: TCCTCACATGGGGGAGGTAG	
*GAPDH*	F: AGGTCGGTGTGAACGGATTTG	123
	R: TGTAGACCATGTAGTTGAGGTCA	

**Abbreviations:** EGFR, epidermal growth factor receptor; GAPDH, Glyceraldehyde-3-phosphate dehydrogenase; JAK2, Janus kinase 2; RT-PCR, reverse transcription–polymerase chain reaction; STAT3, Signal transducer and activator of transcription 3.

## Data Availability

The corresponding author [WM] would gladly provide access to the datasets used and analyzed during this investigation if a fair request is made.

## LIST OF ABBREVIATIONS

ARG: Arctigenin
DMSO: Dimethyl Sulfoxide
EGFR: Epidermal Growth Factor Receptor
FBS: Fetal Bovine Serum
JAK2: Janus Kinase 2
NPC: Nasopharyngeal Carcinoma
RPMI: Roswell Park Memorial Institute
